# A Mixed-Methods Study to Examine the Role of Psychosocial Stress and Air Pollution on Hypertension in Mexican-Origin Hispanics

**DOI:** 10.1007/s40615-018-0490-1

**Published:** 2018-04-20

**Authors:** Amal Rammah, Kristina Walker Whitworth, Inkyu Han, Wenyaw Chan, Maria D. Jimenez, Sara S. Strom, Melissa L. Bondy, Elaine Symanski

**Affiliations:** 1 Epidemiology, Human Genetics and Environmental Sciences, The University of Texas Health Science Center at Houston (UTHealth) School of Public Health, 1200 Herman Pressler Street, Houston, TX 77030 USA; 2 Southwest Center for Occupational and Environmental Health (SWCOEH), The UTHealth School of Public Health, 1200 Herman Pressler Street, Houston, TX 77030 USA; 3 Epidemiology, Human Genetics and Environmental Sciences, The UTHealth School of Public Health, San Antonio Regional Campus, 7411 John Smith Drive, San Antonio, TX 78229 USA; 4 Department of Biostatistics, The University of Texas Health Science Center at Houston (UTHealth) School of Public Health, 1200 Herman Pressler Street, Houston, TX 77030 USA; 5 Department of Epidemiology, University of Texas MD Anderson Cancer Center, 1155 Pressler, Unit 1340, Duncan Building (CPB) 4th floor, Houston, TX 77030 USA; 6 Department of Medicine, Epidemiology and Population Science, Baylor College of Medicine, One Baylor Plaza, Suite 422A, Houston, TX 77030 USA

**Keywords:** Air pollution, Psychosocial stress, Hypertension, Mexican-origin Hispanics

## Abstract

**Purpose:**

Independent and combined effects of air pollution and psychosocial stressors on hypertension, a risk factor for cardiovascular disease, among Hispanics are not well studied.

**Methods:**

We administered a pilot-tested questionnaire on individual- and neighborhood-level psychosocial stressors, developed with community input, to nearly 2500 individuals from the MD Anderson Cancer Center cohort of Mexican-Americans. We used data from local air quality monitors to estimate individual exposures to ozone (O_3_) and fine particulate matter (PM_2.5_) for the 12-month period preceding enrollment using inverse distance interpolation. We applied logistic regression models to examine relationships between exposures to psychosocial stressors and air pollution with prevalent hypertension and used stratified analyses to examine the interacting effects of these two exposures on hypertension_._

**Results:**

There was a positive association between prevalent hypertension and a high frequency of feeling anxious or depressed (prevalence odds ratio (POR) = 1.36, 95% CI [1.06–1.75]) and experiencing aches and pains (POR = 1.29, 95% CI [1.01–1.64]). The odds of having hypertension were also elevated among those worrying about their own health (POR = 1.65, 95% CI [1.30–2.06]) or about not having enough money (POR = 1.27, 95% CI [1.01–1.6]). We observed an inverse association between O_3_ and hypertension. There was no interaction between psychosocial stressors and O_3_ on hypertension**.**

**Conclusion:**

Our findings add to the evidence of a positive association between individual and family stressors on hypertension among Hispanics and other racial/ethnic groups. Contrary to previous studies reporting positive associations, our results suggest that long-term exposure to O_3_ may be inversely related to prevalent hypertension.

## Introduction

A relatively large body of literature has examined associations between psychosocial stressors and hypertension, one of the leading risk factors for cardiovascular disease (CVD). Making comparisons between investigations, however, is challenging. Not only do the specific domains of psychosocial stress that have been evaluated vary between studies, but the indicators selected to measure these domains and the duration (e.g., acute versus chronic) and context (e.g., at work) in which stress occurs also differ [1]. Further, most of the research has focused on non-Hispanic whites [2–5].

Relatively less is known about the impact of psychosocial stress on the risk of hypertension among Hispanics in the United States (U.S.), for whom CVD is the leading cause of death [6]. Gallo et al. found that self-reported chronic stress was positively associated with increased odds of hypertension (OR = 1.10, 95% CI [1.02–1.19]) whereas traumatic stress was associated with a lower odds of hypertension (0R = 0.88, 95%, CI [0.82–0.93]) among adult Hispanics largely from Mexico, Cuba, and Central America [1]. Among postmenopausal Hispanic women, Zambrana et al. reported a positive association between depression and hypertension at baseline (OR = 1.25, 95% CI [1.04–1.51]), as well as between history of depression and pre-hypertensive status (OR = 1.27, 95% CI [1.01–1.61) [7]. Acculturation has also been studied as a psychosocial risk factor for hypertension among Hispanics with conflicting findings [8–11]. Additionally, perceived race-based discrimination has been associated with hypertension among racial and ethnic minorities [12]. Hicken et al. found that racism-related vigilance, a source of chronic stress, is associated with hypertension among Hispanics (OR = 1.05, 95% CI [0.99–1.12) [13]. Further, LeBron et al. report that Latino immigrants are more likely to experience increases in blood pressure associated with individual or institutional discrimination compared to US-born Latinos [14].

Beyond psychosocial stressors, there are ample studies underscoring the putative role of exposures to outdoor air pollutants on risk of hypertension [15–20]. Of particular concern is fine particulate matter (particulate matter with aerodynamic diameter less than 2.5 μm; PM_2.5_) [18, 21–24]. In contrast, fewer studies have examined the risks of hypertension associated with exposure to ozone (O_3_) [16, 18], a secondary air pollutant formed when oxides of nitrogen and volatile organic compounds interact in the presence of sunlight. Thus far, the relation between O_3_ exposure and hypertension is equivocal [25–30] and unlike PM_2.5_, no studies have investigated the association between O_3_ and hypertension among U.S. Hispanics.

Proinflammatory and oxidative stress pathways have been posited as underlying biological mechanisms for CVD. Potential pathways linking psychosocial stressors and CVD involve neuroendocrine activity of the autonomic nervous system (ANS) and the hypothalamus-pituitary adrenal (HPA) axis [31]. Ambient air pollutants are capable of mediating adverse cardiovascular responses through several mechanisms, such as impacting endothelial and other hemodynamic function, triggering acute autonomic imbalance and oxidative stress in the lungs with systematic inflammatory responses [15–20].

We designed a study to address the paucity of literature informing the role of air pollution and psychosocial stress on hypertension among individuals of Mexican-origin in Houston, Texas. In addition to being a busy seaport and home to the largest petrochemical complex in the country, Houston’s heavy traffic contributes to its poor air quality and the city’s diverse residents face documented health disparities [32–35]. Finally, while there is evidence of health inequity linked to myriad neighborhood-level environmental and social factors in urban centers [36, 37], the combined effect of air pollution and psychosocial stressors, particularly among Hispanics, is not well understood. Thus, our overall objective was twofold: (1) using a mixed-methods approach, to assess exposures to ambient air pollution (PM_2.5_ and O_3_) and individual- and neighborhood-level psychosocial stressors in this vulnerable population and (2) to evaluate the independent and interacting effects of these chemical and non-chemical stressors on prevalent hypertension.

## Methods

### Study Population

Participants were randomly selected from The University of Texas MD Anderson Cancer Center (MDACC) Mano a Mano Mexican-American cohort study in Houston, Harris County, Texas (*n* = 23,606) [38]. At baseline, participants complete an interview in the language of their choice (either English or Spanish) and provide information about health status, demographic characteristics, access to healthcare, degree of acculturation, lifestyle behaviors, and occupational and residential histories. As part of the Mano a Mano study, additional follow-up telephone interviews occur every 6 months.

For the present analysis, we recruited 2481 participants aged 20 years or older who enrolled in Mano a Mano between 2007 and 2014, based on responses to the question: “have you been told by a health professional that you have high blood pressure, also called hypertension?” After excluding participants (*n* = 13) without valid geographic coordinates for their residential address, the final sample size was 2468: 1135 cases with hypertension and 1333 controls without hypertension. There were 87 households with two participants and six households with more than two participants.

### Psychosocial Stressors

We collected primary data in 2014–2015 during regularly scheduled Mano a Mano follow-up interviews*.* Following development of a questionnaire [39], trained interviewers administered a 32-item survey in the language preference of participants (English or Spanish) on psychosocial stressors in the home, neighborhood, and at work that they may have experienced at the time they enrolled. Questions were also asked about certain behaviors and lifestyle preferences that might affect exposure to air pollutants. Participants responded to each question using a five-point Likert scale: *Not at all*; *Yes, a little bit*; *Yes, sometimes*; *Yes, a lot of the time*; *Yes, Most of the time*. For analyses, responses were collapsed into three categories: low (*no*, *not at all* and *yes, a little bit*), medium (*yes, sometimes*) and high (*yes, a lot of the time* and *yes, most of the time*). Large proportions of participants (66% percent of cases and 63% of controls) did not answer the questions about work-related stressors (e.g., occupational exposure to chemicals, unsafe work conditions, working too hard). Hence, these questions were not further analyzed.

### Long-Term Exposures to PM_2.5_ and O_3_

**Fig. 1:**
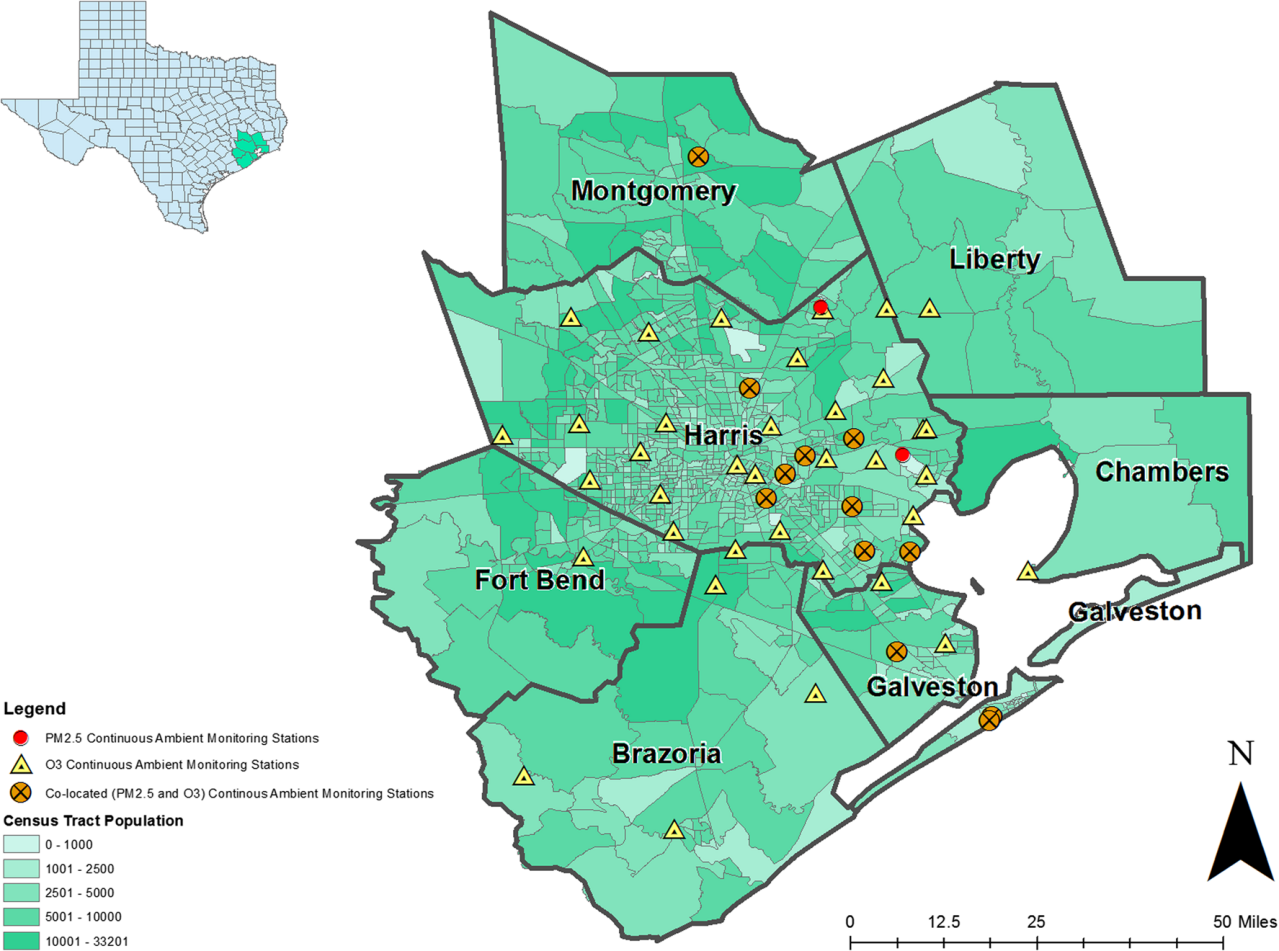
Locations of all regulatory and non-regulatory continuous ambient monitoring stations for O_3_ and PM_2.5_ active at any time in the study region from 2006—2014. The number of O_3_ monitoring stations ranged from 41 to 45 in a given year: 41 in 2013; 42 in 2007, 2008, 2010, and 2014; 43 in 2006 and 2012; 45 in 2009 and 2011. The number of PM_2.5_ stations included was 9 in 2006, 2011–2014; 10 in 2007–2008; and 11 in 2009–2010

We obtained validated hourly air pollution data for O_3_ and PM_2.5_ from the Texas Commission on Environmental Quality (TCEQ), the environmental agency for the state. We used data from all active monitoring stations that continuously measured hourly O_3_ (*n* = 49) and PM_2.5_ (*n* = 15) concentrations in the 8-county greater Houston area (i.e., Brazoria, Chambers, Fort Bend, Galveston, Harris, Liberty, Montgomery, and Waller Counties) for 2006 through 2014 (Fig. 1). We excluded one O_3_ monitoring station and one PM_2.5_ monitoring station reporting ≥ 25% missing observations over the entire study period. Ozone and PM_2.5_ concentrations were reported in parts per billion and micrograms per cubic meter, respectively.

Exposure estimates were constructed using SAS (Version 9.4, SAS Institute, Cary, North Carolina) and ArcGIS Desktop (Release 10.2.2., Environmental Systems Research Institute (ESRI), Redlands, California). We calculated the maximum average eight-hour O_3_ concentration within a 24-h period and the daily average PM_2.5_ concentration for each monitoring station from January 1, 2006 to December 31, 2014. Using this daily time series of O_3_ and PM_2.5_ concentrations, we assigned individual exposure estimates based on the average concentration for the 12-month period preceding baseline enrollment using inverse distance weighting (IDW; *p* = 2) [40] for the three monitoring stations nearest to the participant’s geocoded residential address.

### Covariates

Demographic characteristics were obtained during the baseline Mano a Mano interview. Age at baseline was categorized into four strata (<30, 30–39, 40–39, 50+). Education was assessed in terms of highest level completed and collapsed into three levels (< High School, High School/General Education Development (GED), and > High School education). Nativity status (U.S.- or Mexico-born) was used to measure acculturation. Annual household income in the year preceding baseline was broken down into four levels (≤ $24,999, $25,000 to $44,999, $45,000 to $74,999, ≥ $75,000). Smoking and alcohol use were categorized as current, former, or never. Body Mass Index (BMI) was calculated and categorized as underweight/normal weight (< 25.0 kg/m^2^), overweight (25.0–< 30.0 kg/m^2^), obese (30.0–< 35.0 kg/m^2^), extremely obese II (35.0–< 40.0 kg/m^2^), and extremely obese III (≥ 40.0 kg/m^2^). Having asthma at baseline as diagnosed by a healthcare provider was reported as “yes” or “no”.

### Statistical Analyses

All statistical analyses were conducted using SAS software (Version 9.4, SAS Institute, Cary, North Carolina). We used logistic regression and computed prevalence odds ratios (POR) and 95% confidence intervals (CI) to examine associations between psychosocial stressors or air pollution and hypertension. We examined air pollution exposures as continuous or categorical (quartiles) variables, in separate models. The following variables were identified *a priori* as risk factors and included in all adjusted models: age, sex, nativity, smoking, alcohol, BMI, and having asthma. We also evaluated education and employment using the change-in-estimate approach [41] but their inclusion did not change the effect estimate by more than 10% and thus, they were excluded from the final models. We additionally used stratified analysis to examine potential interaction between air pollution and those psychosocial stressors that were independently associated with hypertension (*p* < 0.05).

### Sensitivity Analyses

We conducted sensitivity analyses using air pollution exposure estimates constructed with a single (i.e., the closest) monitor and applied mixed-effects logistic regression models with household specified as random effect to account for the correlation among individuals living together in the same household.

The Institutional Review Boards at MDACC and the University of Texas Health Science Center at Houston (UTHealth) approved the study and oral informed consent during phone interviews was obtained from all participants.

## Results

The majority of participants lived within the Houston city limits (66.25%). The next largest proportion of participants lived in Pasadena (22.11%), a community located east of Houston, near the Houston Ship Channel and numerous industrial facilities. Individuals ranged in age from 20 to 60 years at baseline; the mean age was 53 years (SD 11.15) among cases and 40 years (SD 10.77) among controls (Table 1). Over 85% of cases and controls were women and most had less than 12 years of education (64% of cases and 57% of controls) and were born in Mexico (72% of cases and 85% of controls). A large proportion of cases (42%) and controls (39%) did not report on income (data not shown).

**Table 1:** Sociodemographic characteristics and self-reported hypertension among Mexican-origin Hispanics (*N* = 2468) (missing observations are not shown), Houston, Texas, 2007–2014

	Cases (*n* = 1135)	Controls (*n* = 1333)	OR	95% CI	
Age (years)					
< 30	22	219	Ref		
30–39	131	503	2.59*	1.61	4.18
40–49	271	359	7.51*	4.72	11.97
50+	711	252	28.09*	17.71	44.55
Gender					
Men	163	99	Ref		
Women	972	1234	0.48*	0.37	0.62
Nativity					
Mexico	820	1131	Ref		
USA	314	201	2.16*	1.77	2.63
Education (years)					
13+	209	264	Ref		
< 12	722	764	1.19	0.97	1.47
High school graduate/GED	203	305	0.84	0.65	1.08
Employment					
Never worked	192	210	Ref		
Ever worked	936	1103	0.93	0.75	1.15
Smoking					
Never	842	1113	Ref		
Current	81	88	1.22	0.89	1.67
Former	211	132	2.11*	1.67	2.67
Alcohol Consumption					
Never	837	1049	Ref		
Current	169	200	1.06	0.85	1.33
Former	129	79	2.05*	1.53	2.75
Body mass index (kg/m^2^)					
Underweight/normal weight (< 24.9)	103	244	Ref		
Overweight (25.0 to 29.9)	303	455	1.58*	1.20	2.07
Obese I (30.0 to 34.9)	309	350	2.09*	1.59	2.76
Obese II (35.0 to 39.9)	199	168	2.81*	2.06	3.82
Obese III (≥ 40.0)	190	75	6.00*	4.22	8.54
Asthma					
No	451	342	Ref		
Yes	684	991	0.52*	0.44	0.62

*OR* odds ratio; *CI* confidence interval

*p < 0.05 for associations between covariates and hypertension

Selected percentiles of the distribution of O_3_ and PM_2.5_ exposures appear in Table 2. The median (IQR) O_3_ exposure was 35.41 ppb (4.78) among cases and 36.07 ppb (2.69) among controls. The median (IQR) PM_2.5_ exposure was 11.44 μg/m^3^ (1.12) among cases and 11.58 μg/m^3^ (0.85) among controls. Due to the lack of variability in estimated exposure to PM_2.5_, we excluded this pollutant from subsequent analyses.

**Table 2:** Distribution of annual 8-h maximum O_3_ and 24-h average PM_2.5_ exposure estimates among Mexican-origin Hispanics *N* = 2468), Houston, Texas, 2007–2014

	Mean ± SD	25th percentile	50th percentile	75th percentile	Range
O_3_ (ppb)					
Cases	35.45 ± 1.95	34.06	35.41	38.84	27.54–42.11
Controls	35.88 ± 2.04	34.56	36.07	37.25	23.77–43.97
PM_2.5_ (μg/m^3^)					
Cases	11.60 ± 0.92	10.94	11.44	12.06	9.51–14.88
Controls	11.77 ± 0.92	11.24	11.58	12.09	9.36–14.92

Table 3 presents adjusted associations between hypertension and sources of psychosocial stress. There was a positive association between both reporting high frequency of stress due to unfair or disrespectful treatment based on race, ethnicity, or immigration status (POR = 1.55, 95% CI [1.04–2.32]) as well as stress due to too much litter or trash in the neighborhood (POR = 1.48, 95% CI [1.06–2.07]) and hypertension. Lower odds of prevalent hypertension were observed among individuals experiencing medium (POR = 0.60, 95% CI [0.40–0.90]) and high (POR = 0.87, 95% CI [0.55–1.38]) levels of stress due to domestic violence.

**Table 3:** Association between sources of psychosocial stressors and prevalent hypertension among Mexican-origin Hispanics (*N* = 2468) (missing observations are not shown) Houston, Texas, 2007–2014

Stressor	Cases (*n* = 1135)	Controls (*n* = 1333)	POR^1^	95% CI	
Domestic violence					
Low	1023	1196	Ref		
Medium	58	85	0.60*	0.40	0.90
High	54	52	0.87	0.55	1.38
Problems with children					
Low	582	728	Ref		
Medium	249	352	1.03	0.82	1.31
High	279	249	1.17	0.92	1.49
Caring for a sick family member					
Low	788	1005	Ref		
Medium	145	156	1.03	0.77	1.37
High	202	172	0.99	0.76	1.30
Separated from family living elsewhere					
Low	866	890	Ref		
Medium	123	226	0.83	0.63	1.10
High	146	217	0.90	0.68	1.19
Contact with authorities/law enforcement					
Low	975	1112	Ref		
Medium	99	147	0.80	0.59	1.10
High	61	74	0.73	0.49	1.10
Unfair treatment/disrespect based on race, ethnicity or immigration status					
Low	179	357	Ref		
Medium	119	213	1.13	0.80	1.59
High	100	89	1.55*	1.04	2.32
Neighborhood noise					
Low	898	1106	Ref		
Medium	141	151	1.14	0.85	1.53
High	96	76	1.19	0.81	1.73
Neighborhood traffic/construction					
Low	749	884	Ref		
Medium	208	272	0.99	0.77	1.26
High	178	177	1.09	0.82	1.43
Neighborhood litter/trash					
Low	871	1070	Ref		
Medium	138	168	0.98	0.73	1.31
High	126	95	1.48*	1.06	2.07
Being safe in home or neighborhood					
Low	716	850	Ref		
Medium	255	340	0.99	0.79	1.24
High	164	143	1.21	0.90	1.62
Unknown people hanging around the neighborhood					
Low	788	922	Ref		
Medium	209	289	0.92	0.72	1.17
High	138	122	1.17	0.85	1.61
Violence at children’s school					
Low	845	956	Ref		
Medium	152	235	0.82	0.63	1.07
High	138	142	1.13	0.84	1.52

*POR* prevalent odds ratio; *CI* confidence interval

*p < 0.05 for associations between covariates and hypertension status

^1^Adjusted for age, sex, nativity, smoking, alcohol consumption, BMI and asthma

Table 4 presents adjusted associations between stress-related conditions and hypertension. There was a positive association between both a high frequency of feeling anxious or depressed (POR = 1.36, 95% CI [1.06–1.75]) and experiencing aches, pains or nausea (POR = 1.29, 95% CI [1.0–1.64]) and hypertension. The odds of having hypertension were also elevated among those with concerns about health (POR = 1.65, 95% CI [1.30–2.06]) or not having enough money (POR = 1.27, 95% CI [1.01–1.6]).

**Table 4:** Associations between stress-related conditions and prevalent hypertension among Mexican-origin Hispanics (*N* = 2468), Houston, Texas, 2007–2014

Stress-related condition	Cases (*n* = 1135)	Controls (*n* = 1333)	POR^1^	95% CI	
Anxiety/depression due to stress					
Low	596	758	Ref		
Medium	277	361	1.00	0.80	1.26
High	262	214	1.36*	1.06	1.75
Aches/pains/nausea due to stress					
Low	594	754	Ref		
Medium	255	333	1.05	0.83	1.33
High	286	246	1.29*	1.01	1.64
Trouble sleeping due to stress					
Low	552	740	Ref		
Medium	228	320	0.93	0.73	1.19
High	355	273	1.22	0.96	1.53
Worrying about not having enough time for oneself					
Low	566	594	Ref		
Medium	226	360	0.80	0.63	1.02
High	343	379	0.96	0.77	1.20
Worrying about one’s own health					
Low	430	658	Ref		
Medium	257	372	1.05	0.82	1.33
High	448	303	1.65*	1.30	2.06
Worrying about not having enough money					
Low	450	518	Ref		
Medium	302	451	1.06	0.84	1.35
High	383	364	1.27*	1.01	1.60

*POR* prevalent odds ratio; *CI* confidence interval

*p < 0.05 for associations between covariates and hypertension status

^1^Adjusted for age, sex, nativity, smoking, alcohol consumption, BMI and asthma

Adjusted PORs (95% CIs) for the association between O_3_ exposure and hypertension were 0.89 (0.69–1.15), 0.44 (0.33–0.58), and 0.55 (0.42–0.72) for the second, third, and fourth quartiles of O_3_, respectively, as compared to the lowest quartile. Odds of hypertension decreased by a factor of 0.90 for each parts per billion increase in exposure to O_3_ (adjusted POR = 0.90, 95% CI [0.86–0.95]). There was no evidence of effect measure modification by psychosocial stress in the association between ozone and hypertension. These results did not change when using data from a single monitor to construct air pollution exposure estimates (data not shown). Further, the results from the mixed-effects models accounting for the correlation among individuals living in the same household were similar as well (Adjusted PORs (95% CIs) were 0.89 (0.68–1.15), 0.44 (0.33–0.59), and 0.55 (0.41–0.73) for the second, third and fourth quartiles of O_3_, respectively).

## Discussion

We examined co-exposures to air pollution and psychosocial stress among an overburdened population, i.e., Mexican-origin Hispanics living in Houston, Texas. We observed elevated odds of prevalent hypertension with several conditions resulting from stress including feeling anxious or depressed, experiencing aches, pains, or nausea and having concerns about poor health and not having enough money. Additionally, we detected associations between hypertension and being unfairly treated or disrespected because of race ethnicity or immigration status and having too much litter and trash in the neighborhood. While we could not examine associations with PM_2.5_ because of too little variability in our exposure estimates, we found inverse associations between ozone exposure and hypertension.

In our study, experiencing a high level of stress-induced anxiety or depression was associated with a 36% increase in the odds of prevalent hypertension. Zambrana et al. reported similar associations between depression and prevalent hypertension among postmenopausal Hispanic women ages 50 and older [7]. In a meta-analysis, Meng et al. reported elevated risks of hypertension with depression, which increased with longer follow-up time [42]. We also found that a high level of stress from unfair or disrespectful treatment based on race, ethnicity or immigration status was positively associated with hypertension, which is consistent with the literature on perceived racial discrimination and hypertension [12–14]. In contrast, we observed that experiencing stress from domestic violence in the home resulted in lower odds of prevalent hypertension, which is similar to the inverse association reported previously between traumatic stressors (including physical or sexual assault) and hypertension [1].

We found an inverse association between 12-month averaged ozone exposure and prevalent hypertension. This finding is consistent with previous studies of short-term [29, 30] but not long-term exposure. Chuang et al. reported a 21.51-mmHg (95% CI [16.90–26.13]) change in systolic blood pressure and a 20.56-mmHg (95% CI [18.14–22.97]) change in diastolic blood pressure with an IQR increase of 8.95 ppb in 1-year averaged O_3_ concentrations among Taiwanese men and women ages 54 and older [27]. A study conducted in China found that an IQR increase of 22 μg/m^3^ (approximately 11 ppb) in 3-year averaged O_3_ concentrations increased the odds of prevalent hypertension (OR = 1.13, 95% CI [1.06–1.20]) [28]. In a study of black women in the U.S., Coogan et al. reported elevated risks of hypertension per IQR increase of 6.7 ppb of averaged O_3_ levels over 2 years (hazard ratio (HR) = 2.09, 95% CI [1.00–1.18]) [43]. The inconsistency between our findings and the results previously reported may be due to differences in the degree of variability in O_3_ exposure in our study population (IQR = 2.75 ppb), particularly, as compared to populations outside of the U.S.

Our study relied on prevalent cases of hypertension and was therefore unable to establish a temporal relationship between the exposures and the outcome. Further, the validity and accuracy of using self-reported hypertension have been evaluated with inconsistent results [44–49]. Hence, it will be important to evaluate the association of psychosocial stress and air pollution with incident hypertension when follow-up data become available in the Mexican-American Mano a Mano cohort. We constructed long-term estimates of O_3_ exposure based on the residential address of each participant using a relatively large air pollution database from stationary monitors in the study region. Thus, our exposure assessment likely captured spatial and temporal influences on outdoor air levels of ozone. Yet, the lack of equally distributed monitors in the study area may have introduced some error in our exposure assessment. Notwithstanding the complex and multi-dimensional aspects of stress that make it difficult to study, a strength of our study was in the use of mixed methods to assess exposure to psychosocial stressors.

Our study provides evidence of positive associations between multiple indicators of psychosocial stress in the family, social and neighborhood environments and hypertension in an ethnically homogenous population of Mexican-origin Hispanics. As CVD remains the leading cause of death among U.S. Hispanics, developing interventions that target some of these potentially modifiable sources of psychosocial stress may lead to improvements in cardiovascular health among this population.
